# Chromosome 15q25 (*CHRNA3-CHRNB4*) Variation Indirectly Impacts Lung Cancer Risk in Chinese Males

**DOI:** 10.1371/journal.pone.0149946

**Published:** 2016-03-04

**Authors:** Yalei Zhang, Mei Jiang, Qin Li, Wenhua Liang, Qihua He, Weiqing Chen, Jianxing He

**Affiliations:** 1 Department of Thoracic Surgery, State Key Laboratory of Respiratory Diseases, The First Affiliated Hospital of Guangzhou Medical University, Guangzhou, P. R. China; 2 State Key Laboratory of Respiratory Disease, National Clinical Research Center for Respiratory Disease, Guangzhou Institute of Respiratory Diseases, The First Affiliated Hospital of Guangzhou Medical University, Guangzhou, P. R. China; 3 Department of Medical Statistics and Epidemiology, The School of Public Health, Sun Yat-Sen University, Guangzhou, P. R. China; Legacy, Schroeder Institute for Tobacco Research and Policy Studies, UNITED STATES

## Abstract

**Introduction:**

Recently, genome-wide association studies (GWAS) in Caucasian populations have identified an association between single nucleotide polymorphisms (SNPs) in the *CHRNA5-A3-B4* nicotinic acetylcholine receptor subunit gene cluster on chromosome 15q25, lung cancer risk and smoking behaviors. However, these SNPs are rare in Asians, and there is currently no consensus on whether SNPs in *CHRNA5-A3-B4* have a direct or indirect carcinogenic effect through smoking behaviors on lung cancer risk. Though some studies confirmed rs6495308 polymorphisms to be associated with smoking behaviors and lung cancer, no research was conducted in China. Using a case-control study, we decided to investigate the associations between *CHRNA3* rs6495308, *CHRNB4* rs11072768, smoking behaviors and lung cancer risk, as well as explore whether the two SNPs have a direct or indirect carcinogenic effect on lung cancer.

**Methods:**

A total of 1025 males were interviewed using a structured questionnaire (204 male lung cancer patients and 821 healthy men) to acquire socio-demographic status and smoking behaviors. Venous blood samples were collected to measure rs6495308 and rs11072768 gene polymorphisms. All subjects were divided into 3 groups: non-smokers, light smokers (1–15 cigarettes per day) and heavy smokers (>15 cigarettes per day).

**Results:**

Compared to wild genotype, rs6495308 and rs11072768 variant genotypes reported smoking more cigarettes per day and a higher pack-years of smoking (P<0.05). More importantly, among smokers, both rs6495308 CT/TT and rs11072768 GT/GG had a higher risk of lung cancer compared to wild genotype without adjusting for potential confounding factors (OR = 1.36, 95%CI = 1.09–1.95; OR = 1.11, 95%CI = 1.07–1.58 respectively). Furthermore, heavy smokers with rs6495308 or rs11072768 variant genotypes have a positive interactive effect on lung cancer after adjustment for potential confounding factors (OR = 1.13, 95%CI = 1.01–3.09; OR = 1.09, 95%CI = 1.01–3.41 respectively). However, No significant associations were found between lung cancer risk and both rs6495308 and rs11072768 genotypes among non-smokers and smokers after adjusting for age, occupation, and education.

**Conclusion:**

This study confirmed both rs6495308 and rs11072768 gene polymorphisms association with smoking behaviors and had an indirect link between gene polymorphisms and lung cancer risk.

## Introduction

Lung cancer is the most common cancer worldwide and accounts for about 23% of the total cancer-related deaths [1]. In 2012 alone, about 0.42 million Chinese males died of lung cancer [2]. Smoking tobacco is a major risk factor for lung cancer; of the Smoking population, more than 80% are at risk for lung cancer [3, 4]. Cigarette smoke contains at least 250 harmful chemicals, more than 50 of which are carcinogens, including polycyclic aromatic hydrocarbons and nicotine metabolites such as 4-(methylnitrosamino)-1(3-pyridyl)-1-butanone (NNK) and N-nitrosonornicotine (NNN) [5]. These nitrosamines form DNA adducts that cause mutations resulting in lung cancer [6]. However, under the same environmental circumstances, only a small fraction of smokers (usually <20%) develop lung cancer; Inter-individual susceptibility to lung cancer may explain this outcome [7].

Nicotinic acetylcholine receptor subunits (nAChRs) belong to the super family of ligand-gated ion channels, and can be activated by nicotine and its metabolites such as NNK and NNN. Nicotine-mediated activation of nAChRs expressed in the key regions of brain can initiate nicotine addiction, thus making individuals susceptible to lung cancer[8,9]. In addition, nAChRs expressed in the alveolar epithelial cells can be activated by nicotine or its metabolites to cause cells’ loss of contact inhabitation and resistance to apoptosis[10–13]. Moreover, the variants in nAChRs may increase individuals’ vulnerability to nicotine and the harmful effects of tobacco smoke [14–16]. The above biologic mechanisms seem a plausible explanation of the associations between SNPs in *CHRNA5-A3-B4* gene clusters, smoking behaviors, and lung cancer risk.

In 2008, three genome–wide association studies (GWAS) in Caucasian populations found three Single nucleotide polymorphisms (SNPs) (rs1051730, rs8034191 and rs16969968) in nicotinic acetylcholine receptor subunit gene cluster (CHRN5-A3-B4) on chromosome 15q25 to be associated with smoking behaviors[14,17,18]. Subsequently, a sea of case-control studies and meta-analyses have verified SNPs in *CHRNA5-A3-B4* play an important role in susceptibility to lung cancer and smoking behaviors in European, American and Asian populations[17,19–21]. However, some SNPs, such as rs16969968, are extremely rare in Asians, and no association was found in relation to smoking behaviors and lung cancer in Chinese[19], these results reiterated underscored the differences in genetic markers among different ethnic populations[22,23]. In addition, there is currently no consensus on whether SNPs in CHRN5-A3-B4 have a direct or indirect carcinogenic effect through smoking behaviors on lung cancer risk [17,19–21,24].

These previous studies encouraged us to investigate the associations between other SNPs in *CHRNA5-A3-B4*, smoking behaviors, and lung cancer risk in the Chinese population. Although some previous studies confirmed an association between rs6495308 polymorphisms, smoking behaviors, and lung cancer risk, no research was conducted in China. We, therefore, examined 10 SNPs including rs6495308 (MAF (Minor Allele Frequency) > 0.1 in Asians) in *CHRNA5-A3-B4* gene clusters According to the HapMap data and previous studies[14,17,20,25–27], and, after our initial results, conducted a detailed analysis of rs6495308 and rs11072768 polymorphisms, and explored whether the two SNPs have a direct or indirect effect through smoking behaviors on lung cancer using a case-control study of 1025 patients: 204 with lung cancer and 821 healthy controls.

## Methods

### Study subjects

A community-based case-control study consisting of 204 male lung cancer patients and 821 healthy men was conducted. Patients who were newly diagnosed with cytological or histologically confirmed lung cancer were recruited from The First Affiliated Hospital of Guangzhou Medical University in China from May to October 2013. Controls consisted of healthy male subjects from a chronic disease epidemiology study conducted in Guangzhou and Zhuhai China from July 2006 to June 2007. All eligible subjects were of the Chinese Han population. In the present study, all subjects have no other cancers or occupational carcinogen exposure history. Ethics Committee of The First Affiliated Hospital of Guangzhou Medical University approved this study.

### Data collection

Trained medical students used a structured questionnaire to acquire socio-demographic characteristics (e.g., age, income, marriage and occupation), complete clinical information such as family history of lung cancer and smoking behaviors via face to face interview. Data regarding histological classification and clinical stage of Lung cancer were obtained by our hospital case management system. 2 ml of venous blood was collected from all subjects for DNA extraction and genotyping, and stored at -80°C until use. Written informed consent was obtained from all patients and controls for the use of their DNA and clinical information.

### Measures of cigarette smoking

A patient who has smoked more than 100 cigarettes in one’s life-time or smoked at least one cigarette per day for more than one year was referred as a ‘smoker’ [4]. ‘Cigarettes per day (CPD)’ was defined as average number of cigarettes smoked per day [4]. ‘Pack-years of smoking’ was counted by dividing 20 from daily cigarette consumption and multiplied duration of smoking among smokers [4]. All subjects were categorized into three groups according to their smoking quantity: never smokers (0 cigarettes/day), lighter smokers (1–15 cigarettes/day), and heavier smokers (>15 cigarettes/day).

### DNA extraction and genotyping

Genomic DNA was extracted from venous blood using a commercial blood DNA kit according to the manufacturer’s instructions (TaKaRa, Dalian, CA, China).The selected SNPs were genotyped using the SNaPshot SNP parting technology (Life technology, Carlsbad, CA, USA). Primers for polymerase chain reaction (PCR) and single-base extension were designed using Assay Designers software version 3.1 (Sequenom, San Diego, CA, USA). Firstly the SNPs were amplified by Multiple PCR reaction using HotStarTaq DNA polymerase (TIANGEN, Beijing, CA, China). Next, genomic amplification products were amplified again using SNaPshot Multiplex extension reactions kit (ABI, Carlsbad, CA, USA) after Purification by shrimp alkali enzyme (Promega, Beijing, CA, China) and external enzyme (Epicentre, Beijing, CA, China). Finally genomic amplification products were assessed by 3730xl genetic analyzer (ABI, Carlsbad, CA, USA). SNP Genotypes were completed by GeneMapper4.1 (ABI, Carlsbad, CA, USA). Genotyping was performed by Commercial genetic testing company (Genesky, Shanghai, CA, China). *CHRNA3* rs6495308 genotypes were categorized into homozygous wild-type (CC), hybrid variant type (CT) and homozygous variant (TT). rs11072768 in *CHRNB4* was classified into homozygous wild-type (TT), hybrid variant type (GT) and homozygous variant (GG). For quality control, 5% of the samples were randomly selected and re-genotyped for all of the selected genes, and the results were 100% concordant.

### Statistical analysis

In this study, either the Chi Square tests (χ2 tests) or the Fisher’s Exact test, whichever appropriate, were used to analyze the differences in the distribution of general demographic characteristics and CPD between cases and controls, Subsequently, Kruskal-Wallis test was conducted to assess the association of rs6495308 and rs11072768 polymorphisms with smoking behaviors. We adjusted the significance for multiple comparisons using the Bonferroni correction. Finally, a series of unconditional logistic regression binary logistic regression analyses was carried out to evaluate the associations between rs514743 genotypes, rs11072768 genotypes, smoking behaviors and lung cancer. Hardy–Weinberg distribution testing was performed for rs6495308 and rs11072768 among controls. For all tests, a two sided *P*<0.05 was considered statistically significant, and all statistical analysis were performed on SPSS version 19.0 software package (SPSS, Chicago, IL, USA).

## Results

### Comparison of demographic characteristics between case and control groups

There were statistically different distributions of age, daily cigarette consumption, occupation, education, marriage and the allele distribution of these two SNPs between cases and controls. More details are presented in Table 1.

**Table 1:** Comparison of demographic characteristics between case and control groups.

Characteristics	Cases	Controls	χ2	*P* value
	(n = 204) (%)	(n = 821) (%)		
Age group			82.79	<0.001
20–29	3(1.5)	41(5)		
30–39	4(2)	127(15.5)		
40–49	28(13.7)	166(20.2)		
50–59	57(27.9)	278(33.9)		
60–69	77(37.7)	162(19.7)		
70–81	35(17.2)	47(5.7)		
Daily cigarette consumption			150.54	<0.001
0 cigarettes/day	45(22.1)	302(36.8)		
1–15 cigarettes/day	18(8.8)	316(38.5)		
>15 cigarettes/day	141(69.1)	203(24.8)		
Smoking pack years	44.89±38.38	13.57±18.59	11.33	<0.001
Occupation			76.46	<0.001
Worker	24(11.8)	229(27.9)		
Farmer	38(18.6)	71(8.6)		
Person in charge	2(1)	42(5.1)		
Technician	9(4.4)	49(6)		
Service personnel	33(16.2)	118(14.4)		
Retired personnel	83(40.7)	171(20.8)		
Jobless	11(5.4)	101(12.3)		
Other	4(2)	40(4.9)		
Education			25.6	<0.001
Illiteracy	7(3.4)	52(6.3)		
Elementary school	50(24.5)	105(12.8)		
Junior middle school	66(32.4)	230(28)		
Senior middle school	56(27.5)	264(32.2)		
College or above	25(12.3)	170(20.7)		
Familial history of cancer				
Yes	30(14.7)	78(9.5)	4.697	0.030
No	174(85.3)	706(90.5)		
Marriage			14.15	<0.001
Yes	195(95.6)	706(86.0)		
No	9(4.4)	115(14.0)		
**rs6495308**				
C/C	87(42.6)	415(50.5)	4.152	0.125
C/T	98(48.0)	336(40.9)		
T/T	19(9.3)	70(8.5)		
**rs11072768**				
T/T	110(53.9)	484(59.0)	2.014	0.365
G/T	85(41.7)	298(36.3)		
G/G	9(4.4)	39(4.8)		

**Fig 1:**
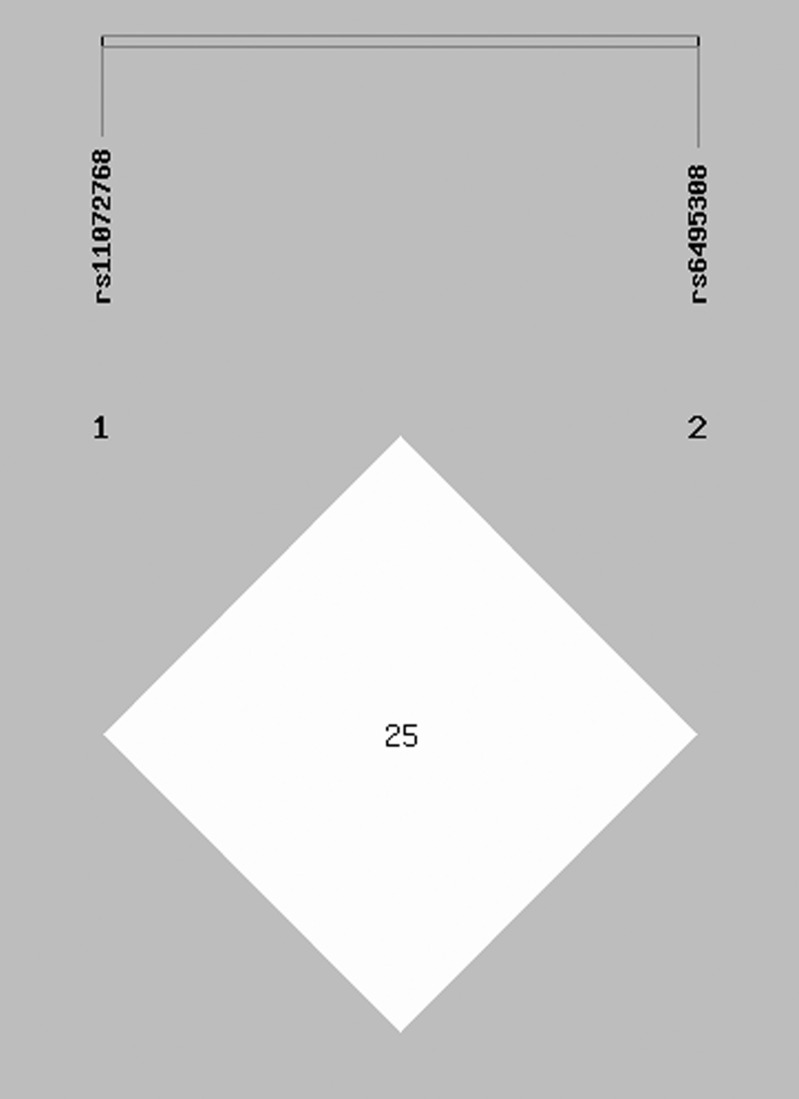
Linkage disequilibrium (LD) patterns of the two SNPs in 15q25.1. Numbers inside the boxes represent *r*^2^ values for LD. Colors indicate the strength of LD between pair-wise combinations of SNPs (white, low LD; red, high LD).

At the same time, we examined the linkage disequilibrium (LD) between rs6495308 and rs11072768 in our data. The LD structure (rs6495308/rs11072768) showed low correlation (*r*^*2*^ = 0.251) (Fig 1).

### Association between gene polymorphisms and smoking behaviors among smokers of lung cancer cases and controls

All subjects were genotyped for two SNPs (rs6495308 C/T, rs11072768 T/G) in 15q25. The SNPs were in Hardy-Weinberg equilibrium (HWE) both in controls and in cases (p>0.05). The associations of rs6495308 and rs11072768 polymorphisms with smoking behaviors were analyzed in 159 smokers with lung cancer and 519 smokers in the control group. The values of cigarettes per day and pack-years were regularly used as the measurements of smoking behaviors for this analysis and can be seen in Table 2. Compared with the wild genotype, rs6495308 and rs11072768 variant genotypes reported smoking more cigarettes per day and had a higher pack-years of smoking among smokers in both groups (P<0.05).

**Table 2:** Association between gene polymorphisms and smoking behaviors among smokers of lung cancer cases and controls. Data were expressed as mean±standard deviation.

Genotype	n(%)	case				control			
		CPD	P	Pack-years	P	CPD	P	Pack-years	P
rs6495308			**0.039**		**0.027**		**0.060**		**0.047**
CC		25.31±8.26		52.75±20.79		13.25±8.06		20.52±17.28	
CT		28.92±15.54*		57.02±35.38*		14.19±8.45		21.17±20.00	
TT		29.62±16.21*		59.11±35.23*		15.02±10.33*		22.05±19.29*	
rs11072768			**0.044**		**0.045**		**0.037**		**0.050**
TT		22.14±7.56		48.04±22.52		14.39±9.99		20.75±16.43	
GT		28.75±15.25^#^		56.05±33.95^#^		14.47±8.29		21.68±21.18	
GG		29.71±15.80^#^		60.70±35.16^#^		14.81±6.98^#^		24.47±19.80^#^	

*: Compared with CC group, p<0.05.

^#^: Compared with TT group, p<0.05.

### Association between gene polymorphisms and lung cancer risk in non-smokers

Table 3. presents the results of associations between rs6495308 and rs11072768 genotypes and lung cancer risks in non-smokers. No significant associations were found between rs6495308 and rs11072768 genotypes and lung cancer risk after adjusting for age, occupation, and education.

**Table 3:** Association between gene polymorphisms and lung cancer risk in non-smokers.

Variables	Cases	Controls	OR (95%CI)^a^	P-value	OR (95%CI) ^b^	P-value
	(n = 45) (%)	(n = 302) (%)				
rs6495308						
CC	19(42.2%)	152(50.3%)	1	0.727	1	0.368
CT	21(46.7%)	120(39.7%)	1.30(0.68–2.49)	0.427	1.93(0.89–3.95)	0.238
TT	5(11.1%)	30(9.9%)	1.20(0.33–4.41)	0.780	1.54(0.42–6.56)	0.632
CT/TT	26(57.8%)	150(49.7%)	1.29(0.69–2.41)	0.131	1.35(0.58–2.50)	0.512
rs11072768						
TT	25(55.6%)	173(57.2%)	1	0.367	1	0.723
GT	17(37.8%)	114(37.8%)	1.59(0.84–3.04)	0.157	1.65(0.69–3.24)	0.318
GG	3(6.7%)	15(4.8%)	1.25(0.27–5.90)	0.777	1.39(0.18–6.58)	0.894
GT/GG	20(33.3%)	129(42.7%)	1.56(0.83–2.92)	0.169	1.54(0.70–3.12)	0.325

^a^OR value unajust for any confoundings

^b^OR value adjustment for age, education, education and marriage and familial history of cancer.

### Association between gene polymorphisms and lung cancer risk in smokers

Associations between gene polymorphisms and lung cancer risk in smokers are shown in Table 4. Among smokers both rs6495308 CT/TT and rs11072768 GT/GG had a higher risk of lung cancer compared to wild genotype without adjusting for potential confounding factors (OR = 1.36, 95%CI = 1.09–1.95; OR = 1.11, 95%CI = 1.07–1.58 respectively). However, no significant associations were confirmed between rs6495308 and rs11072768 genotypes, and lung cancer risk after adjusting for CPD, age, occupation, and education.

**Table 4:** Association between gene polymorphisms and lung cancer risk in smokers.

Variables	Cases	Controls	OR (95%CI) ^a^	P-value	OR (95%CI) ^b^	P-value	OR (95%CI)^c^	P-value
	(n = 159)	(n = 519) (%)						
rs6495308								
CC	68(42.8%)	263(50.7%)	1	0.021	1	0.297	1	0.812
CT	77(48.4%)	216(41.6%)	1.40(1.02–2.03)	0.050	1.39(0.92–2.10)	0.120	1.19(0.68–1.95	0.628
TT	14(8.8%)	40(7.7%)	1.21(0.65–2.26)	0.545	1.22(0.61–2.42)	0.577	1.30(0.50–2.85)	0.596
CT/TT	91(57.2%)	256(49.3%)	**1.36(1.09–1.95)**	**0.049**	1.20(0.77–1.86)	0.420	1.25(0.68–1.88)	0.583
rs11072768								
TT	85(53.4%)	311(60.0%)	1	0.047	1	0.799	1	0.983
GT	68(42.7%)	185(35.6%)	1.13(0.78–1.64)	0.056	1.14(0.76–1.71)	0.527	1.19(0.67–1.85)	0.716
GG	6(3.8%)	24(4.6%)	0.92(0.39–2.19)	0.849	0.95(0.37–2.46)	0.914	1.00(0.25–3.01)	0.998
GT/GG	74(46.5%)	213(40.0%)	**1.11(1.07–1.58)**	**0.027**	1.12(0.75–1.66)	0.579	1.16(0.65–1.82)	0.710

^a^OR value unajustment for any confoundings

^b^OR value adjustment for age, education, education and marriage.

^c^OR value adjustment for age, education, education, marriage daily cigarettes consumption, familial history of cancer and smoking pack years.

### Interaction between gene polymorphisms and smoking behaviors on lung cancer risk

After adjustment for potential confounding factors, we found that the interactions between CPD and both rs6495308 and rs11072768 genotypes were statistically significant: heavy smokers with rs6495308 or rs11072768 variant genotypes were more likely to have lung cancer than respective light smokers with wild genotypes (OR = 1.08, 95%CI = 1.02–3.26; OR = 1.05, 95%CI = 1.01–3.68 respectively), More details are presented in Table 5.

**Table 5:** Interaction between gene polymorphisms and smoking behaviors on lung cancer risk.

Variables	Cases	Controls	OR (95%CI) ^a^	P-value
	(n = 204) (%)	(n = 821) (%)		
CPD				
1–15			1	0.008
>15			9.28(5.86–19.12)	
rs6495308				
CC	87(42.6%)	415(50.5%)	1	0.798
CT	98(48.0%)	336(40.9%)	1.08(0.70–1.96)	0.632
TT	19(9.3%)	70(8.5%)	1.23(0.54–2.88)	0.594
CT+TT	117(57.3%)	406(49.4%)	1.25(0.64–1.95)	0.562
rs11072768				
TT	110(53.9%)	484(59.0%)	1	0.961
GT	85(41.7%)	298(36.2%)	1.19(0.67–1.81)	0.683
GG	9(4.4%)	39(4.8%)	1.04(0.36–2.93)	0.999
GT+GG	94(46.1%)	337(41.0%)	1.08(0.73–1.97)	0.717
interaction(rs6495308×CPD)				
CT/TT×>15			1.08(1.02–3.26)	**0.038**
interaction(rs11072768×CPD)				
GT/GG×>15			1.05(1.01–3.68)	**0.045**

^a^OR value adjustment for age, education, education, marriage, familial history of cancer and smoking pack years.

## Discussion

To our knowledge, this is the first time an investigation on the association between rs6495308, rs11072768 genotypes, smoking behaviors and lung cancer risk in a Chinese male population has been performed. We found rs6495308 and rs11072768 variant genotypes reported smoking more cigarettes over a long period. More importantly, rs6495308 and rs11072768 variant genotypes have a higher risk of lung cancer without adjustment for any potential confounding factors, and heavy smoking has a positive interactive effect with rs6495308 and rs11072768 variant genotypes on lung cancer among smokers. However, no significant associations were found between rs6495308, rs11072768 genotypes and lung cancer risk among nonsmokers and smokers after adjusting for CPD and other potential confounding factors. In this study, a series of results have provided strong evidence that rs6495308 and rs11072768 gene polymorphisms have an indirect impact on lung cancer through smoking behaviors, and there is a correlation between variants in 15q25 and smoking on lung cancer.

A fair amount of research supports the above results. A GWAS meta-analysis in a total sample of 41,150 individuals identified rs6495308 variants in *CHRNA3* increased smoking quantity[28], subsequently, a cohort study confirmed carriers of the rs6495308 TT genotypes have approximately two fold greater odds for ND defined using CPD in European-American smokers[29], these findings suggest that rs6495308 variants lead indirectly to lung cancer via smoking behavior. Furthermore, large GWAS meta-analyses confirmed that the strongest genetic contribution to smoking-related traits comes from variation in *CHRNA5-A3-B4*[10,15,17,30,31], as first revealed on a genome-wide significant level by Thorgeirsson et al. in a study of over 13,000 smokers from Iceland[15]. In addition, a GWAS study in the Chinese population speculated that one non-synonymous mutation in the rs6495308c risk allele results in a higher *CHRNA3* receptor production which may make individuals more sensitive to nicotine and thus more susceptible to nicotine dependency, a well-established etiological factor for lung cancer [19]. Li et al found that the G allele of rs11072768 in *CHRNB4* was significantly associated with smoking initiation (SI) (P = 0.001; OR = 1.22;95%CI: 1.08, 1.37), smoking quantity (SQ) (P = 0.016; OR = 1.16; 95%CI: 1.03, 1.31), and smoking cessation (SC) (P = 0.01; OR = 1.18; 95%CI: 1.04, 1.34) in a sample of Korean males [32]. Other studies have affirmed some SNPs (rs12914385, rs8042374 and rs588765) to be associated with smoking behaviors and are in complete agreement with an indirect link between genotypes and lung cancer[24,27].Nevertheless, there is much assertion that, rather than being an agent for smoking, *CHRNA5-A3-B4* variants have a direct impact on lung cancer risk. Lips et al. calculated a 1.2 difference in CPD due to the fact that rs16969968 homozygotes in *CHRNA5* only contribute a 9% increase in lung cancer risk, which cannot account for the association between the variants, smoking behavior and lung cancer[31]. Hung et al. also indicated an increased risk for lung cancer, even among non-smokers, due to rs16969968 variants, in addition to other evidence suggesting that rs16969968 variant genotypes are not directly associated with an increase in the risk for other smoking-related cancers such as head and neck cancer [33]. Moreover, other studies confirmed an over-representation of *CHRNA5-A3-B4* variants in familial lung cancer cases indicating a direct impact of variants on lung cancer [34]. These discrepancies in the previous studies can most probably be attributed to genetic, racial and environmental differences [14,18,24].

The specific mechanisms of the genetic variations in *CHRNA5-A3-B4* that influenced smoking behaviors were unclear, but there was a potentially plausible biologic hypothesis underlying the observed results. The variants in *CHRNA5-A3-B4* give rise to the over expression of the nAChRs in in the key regions of brain which may make individuals sensitive to nicotine, which includes the pharmacological effects of nicotine along with higher activation of the medial habenula and reduced activation of dopaminergic neurons after acute nicotine administration[35,36], and thus lead to smoking more cigarettes over a long period, a well-established etiological factor for lung cancer. The mechanism indicates the presence of a smoking-by-variant interaction, and such effect is only observed in smokers.

Several limitations of this study should be mentioned. Firstly, self-reported smoking behaviors may be prone to recall bias which influences the authenticity of associations between SNPs, smoking behavior and lung cancer. Secondly, the relatively small sample size could occasionally affect the results of this study. Next, the average age of the case group was higher than that of the control group. In the case group, the largest proportion were retirees, retirees may be higher risk of lung cancer due to their older age; in addition, those with higher education in the case group was lower than that of the control group, those with a higher degree may pay more attention to and be more knowledgeable in regards to their own health condition and, as a result, have relatively fewer adverse health behaviors. Finally, the functional study furtherly cannot be implemented because rs6495308 and rs11072768 are located on intron in 15q25, so there may be other causal SNPs in highly linkage disequilibrium (LD) with the two SNPs within 15q25 region to affect the function of nAchRs. Therefore, a large population-based study is still required to confirm the present findings.

In conclusion, the results of our study confirmed two SNPs (rs6495308 and rs11072768) in *CHRNA5-A3-B4* have a indirect effect on lung cancer through smoking behaviors, and a positive correlation between CPD and both rs6495308 and rs11072768 on lung cancer among smokers. These findings extend our understanding of the possible mechanism of cigarette smoking on lung cancer and may have applications in healthcare for tailoring strategies of smoking cessation

## Supporting Information

S1 Information Original data for the manuscript. (XLS)

S2 Information Annotation of the original data. (DOCX)

S1 Data (SAV)
