# Supplementary material for: Chromosome 15q25 (CHRNA3-CHRNB4) Variation Indirectly Impacts Lung Cancer Risk in Chinese Males
Source: PLoS One. 2016 Mar 4;11(3):e0149946. doi: 10.1371/journal.pone.0149946 (PMC4778880; doi:10.1371/journal.pone.0149946)
Supplement: S2 Information — (DOCX) [file pone.0149946.s002.docx]

1. Case/control 0=control group 1=case group
2. Code the serial number of the control or case group
3. Gender 0=female 1=male
4. Age the age of each Study subject(years)
5. Nationality 1=Han nationality
6. Age 1(years) 1=20-29 2=30-39 3=40-49 4=50-59 5=60-69 6=70-81
7. Occupation 1=worker 2=farmer 3=soldier 4=Person in charge

5=Technician 6=Service personnel 7=housewife

8=Retired personnel 9=Jobless 10=student 11=others

1. Education 1=Illiteracy 2=Elementary school 3=Junior middle school

4=Senior middle school 5=College or above

1. Marriage 1=Unmarried or other 2=married
2. Avrsmn 0=0 cigarettes/day 1=1-15 cigarettes/day 2=>15 cigarettes/day
3. Pack-years mean Smoking pack years
4. CHRNB4rs11072768 1=T/T 2=G/T 3=G/G
5. CHRNA3rs6495308 1=C/C 2=C/T 3=T/T
6. Familial history of cancer 0=No 1=Yes
